# Impulsivity, inhibition, and internet addiction in medical students of North of Iran

**DOI:** 10.3389/fpsyt.2022.1002625

**Published:** 2023-01-19

**Authors:** Mohammad Salehi, Zeinab Abbaspour, Aliasghar Molana, Najmeh Shahini

**Affiliations:** ^1^Golestan Research Center of Psychiatry, Golestan University of Medical Sciences, Gorgan, Iran; ^2^Student Research Committee, Golestan University of Medical Sciences, Gorgan, Iran

**Keywords:** impulsivity, internet addiction, medical student, Go/No-Go, Barratt Impulsiveness Scale (BIS), inhibition, Iran, Young questionnaire

## Abstract

**Background:**

Internet addiction, a serious psychological disorder, has rapidly increased among students and caused substantial interpersonal impairments. On the other hand, some evidence has indicated that impulsivity is associated with addictive behaviors.

**Objectives:**

There are a few studies concerning the relationship between internet addiction and impulsivity in Iranian students. Therefore, this study aimed to evaluate the relationship between impulsivity and internet addiction in the medical students of Golestan University of Medical Sciences, Golestan, Iran, within 2020–2021.

**Methods:**

In a cross-sectional study, 260 medical students at Golestan University of Medical Sciences in 2021 completed demographic, Barratt Impulsiveness Scale, and Young questionnaires and participated in a Go/No-Go computer test. Based on Young test scores, the students were divided into three groups of normal users, at-risk users, and users requiring treatment and compared regarding impulsivity and inhibition ability. Data was analyzed using SPSS v 23.

**Results:**

The mean age of the participants was 24.16 years. The results showed a significant correlation between impulsivity and internet addiction (*p* = 0.001), which was established in all contextual indicators. A significant correlation was also shown between impulsivity and internet addiction (*r* = 0.602 and *p* = 0.001), both in male and female student. However, restraint index was correlated with internet addiction only in females (*r* = 0.187 and *p* = 0.033).

**Conclusion:**

Based on these findings, impulsivity increases alongside the rising of the internet addiction severity and does not influence by gender.

## 1. Background

Communities worldwide are experiencing significant changes in digital technology use (1, 2). Most individuals use the internet as an effective tool in their personal or business life because it provides the possibility of entertainment, communication, and desirable management of daily activities and positively facilitates numerous activities. Internet World Stats indicated that 69.0% of the world's population used the internet in 2022, an increase of 14% since 2000. Increasing the number of internet users worldwide is one of the important factors affecting internet addiction (3). In this regard, internet addiction disorder (IAD), a problematic internet use (PIU) or an inability to control the use of the internet, has become an important issue related to physical and mental health (1, 4, 5).

Internet addiction can have many side effects, including interpersonal, social, occupational, psychological, and physical functioning (1, 6). Internet overuse may lead to physical problems, including less exercise, carpal tunnel syndrome, musculoskeletal pain, dry eyes, fatigue and poor sleep quality, and even self-harm/suicidal behavior (7). On the other hand, psychiatric symptoms might lead to the onset of persistent IAD, which accelerates psychiatric symptoms (8). Previous studies on internet addiction in adults have examined the accompanying psychological variables, such as shyness, loneliness, self-awareness, anxiety, depression, and interpersonal relationships. Moreover, personality traits are identified as one of the important intrinsic factors of addictive behavior, as there is some evidence indicating that internet addiction can be conceptualized as an impulse control disorder and that trait impulsivity is a marker for vulnerability to Internet addiction (1, 9–11). However, the underlying mechanisms of the phenomenon are not clearly defined (8).

Impulsivity includes human behaviors without sufficient thought, instinctual actions without ego, and rapid actions of the mind without foresight and conscious judgment. It has a multidimensional structure, including several dimensions: emphasis on the present, inability to delay reward, sensitivity, risk-taking, inability to conceive and plan, impatience, pleasure-seeking, and sensitivity to reward. Impulsivity might also mean acting with the slightest thought about future behaviors by acting on thoughts that are not the best choice of one or the other (12–15). Impulsive individuals are less able to control internet use; therefore, impulsivity might be a risk factor for IAD and is a sign of vulnerability to IAD.

## 2. Objectives

Based on a meta-analysis of 30 studies and a sample size of 130,531, the growth rate of internet addiction based on the random effects model was 20% in Iran from 2006 to 2015 (16). Moreover, the student population is more vulnerable among internet users because of educational needs, information, entertainment, and more free time to use the Internet (4, 17). Excessive internet use is associated with attention-deficit hyperactivity disorder (ADHD) (18), resulting in poor academic performance (19). In this regard, the necessity of identification, treatment, and prevention of the age groups which are at risk is being sensed by the responsible and related authorities. Since Iranian college students are a less studied target group in this field, this study was designed to determine the relationship between impulsivity and internet addiction in the medical students of Golestan University of Medical Sciences, Golestan, Iran.

## 3. Methods

### 3.1. Study design

The present study was designed and conducted based on a descriptive-cross-sectional method with a pivotal sampling method.

### 3.2. Study population

The sample size of 260 individuals was calculated. First, the list of medical students of Golestan University during 2021–2022 was obtained from the university, and the subjects were randomly selected using a gender ratio of 130 male and 130 female students using random numbers. After explaining the plan to the students, attracting their cooperation, and obtaining informed consent, all students underwent three tests. Due to the COVID-19 pandemic, a questionnaire was completed online. In the present study, three questionnaires were used to collect information in addition to a demographic checklist.

### 3.3. Sample size calculation

According to a study conducted by Yücens and Üzer, with a confidence level of 95%, a prevalence of 27% for Internet addiction in students, and an acceptable error of 5%, the sample size of 260 people was calculated (8).

### 3.4. Data collection instruments

#### 3.4.1. Young's diagnostic questionnaire for internet addiction test

This questionnaire consists of 20 items with six factors, including the importance of the Internet, heavy use, neglect of work, anticipation, lack of control, and neglect of social life (20, 21). Individuals answer the items based on a 5-point Likert scale (never = -, rarely = 1, occasionally = 2, high = 3, often = 4, always = 5). User status is measured based on points (20–49: normal internet use, 50–79: mild internet addiction, 80–100: severe internet addiction). The reliability of Cronbach's alpha coefficient was determined to be 0.88. Content and convergent validity, retest (*r* = 0.82), internal consistency (*a* = 0.88), and bisection (*r* = 0.72) were calculated, which were acceptable according to the results. The best clinical cut-off point of this questionnaire was 46 (22–25).

#### 3.4.2. Barratt Impulsiveness Scale questionnaire

The Barratt Impulsiveness Scale (BIS) will be used to assess the degree of impulsivity. This tool consists of 30 items that have been prepared and scored based on a 4-point scale (never = 1, sometimes = 2, often = 3, almost always = 4) and includes three cognitive subscales with eight factors (indicating complexity and resistance regarding immediate decision-making), a motor subscale with ten factors (representing action without thought and reflection), and an unplanned subscale with 12 factors (disregarding for futurism in behaviors and actions). Respondents whose average total score is 64 or higher are considered impulsive. The lowest and highest scores are 30 and 120, respectively, with a reported total score range of 79–83. The alpha coefficients of cognitive, motor and unplanned impulsivity factors are 0.70, 0.67, and 0.80, respectively. The reliability of the whole test is 0.83 (26).

#### 3.4.3. Go/No-Go test (answer barring)

This software test, widely used to measure behavioral inhibition, includes two categories of stimuli. Subjects should respond to some of these stimuli (go) and refrain from responding to the other group. Because the number of stimuli (go) is usually higher than the number of stimuli (do not), the individual is more willing to respond. Lack of proper inhibition or committing error means performing a motor response when presenting a non-target stimulus. In this test, the stimulus (go) is in the geometric shape of a triangle, which is presented among other geometric shapes (go) in the middle of the 86-inch monitor screen at a distance of 62 cm from the subject's eye for 922 milliseconds. After watching, the subject should respond to it as fast as possible by pressing the space button on the keyboard and should not respond in case of watching other geometric shapes. At first, several attempts are presented as a practice so that the subject is fully acquainted with the test and the placement of the answer key; then, 822 main attempts are presented, 22 of which were “go” stimuli to create a strong answer. All subjects' responses and reaction times are recorded and reported as a presentation error score and deletion error. The total reliability is 0.89. The reliability coefficients of this test were reported between 0.72 and 0.78 (27).

### 3.5. Data analysis

Statistical analysis was performed using SPSS software (version 24). The quantitative data were described as mean ± standard deviation, and the qualitative data were illustrated as frequency and percentage. In analysis, the Kolmogorov–Smirnov test was used to assess normality. The independent *t*-test was used in the case of normality, and the Mann-Whitney *U*-test was utilized in the absence of normality assumptions. The Chi-square test was used to compare the frequency of classified variables. In addition, a one-way analysis of variance was used to compare quantitative variables at different levels of internet addiction. In the absence of normality, the Kruskal-Wallis test was used. Multivariate linear regression was applied to estimate the relationship between independent variables and dependent variable (Internet addiction). The significance level of the tests was considered 0.05.

## 4. Results

Demographics are summarized in Table 1. The mean age of the participants, including 130 males and 130 females, was 24.16 years. Regarding marital status, 226 (87%) students were single, and the rest were married. Based on the Young IAT score, 48 participants (18.4%) indicated low Internet addiction and nine people (3.4%) were in high risk.

**Table 1:** Demographic characteristics in medical students of North of Iran.

**Characteristics**	**Number (%)/mean ±SD**
**Gender**	
Male	130 (50%)
Female	130 (50%)
Age (year)	24.16 ± 1.80
**Marital status**	
Single	226 (87%)
Married	34 (13%)
Young score	41.50 ± 13.92
Normal	204 (78.2%)
Low addiction	48 (18.4%)
High addiction	9 (3.4%)
Go/No-Go	24.03 ± 12.92
Barratt score	76.25 ± 12.99

The mean score of Young and Barratt score in male students was significantly higher than in female students (*p* = 0.003 and *p* = 0.015, respectively). However, these scores were similar in single and married individuals (*p* = 0.070 and *p* = 0.929, respectively). Although the mean Go/No-Go score (inhibitory power) was similar in male and female subjects (*p* = 0.172), single participants indicated higher scores than married people (*p* < 0.001). There was also a significant inverse relationship between age and Go/No-Go score (inhibition power) (*r* = −0.247 and *p* < 0.001) (Table 2).

**Table 2:** Mean scores of relationship between demographic characteristics with Barratt score, Young score, and inhibition power status.

**Characteristics**	**Sex**			**Marital status**			**Age** ^*^	
	**Male**	**Female**	***P***	**Single**	**Married**	***P***	**CC** ^**^	***P***
Internet addiction^*^	44.05 ± 15.56	38.97 ± 11.59	**0.003**	41.68 ± 13.58	40.26+ ± 16.22	0.07	−0.034	0.587
Impulsivity^*^	78.22 ± 14.21	74.29 ± 11.40	**0.015**	76.26 ± 13.13	76.06 ± 12.25	0.929	−0.021	**0.735**
Inhibitory score^*^	25.26 ± 12.84	22.82 ± 12.93	0.172	25.19 ± 13.06	16.32 ± 8.79	**< 0.001**	−0.247	**< 0.001**

* Mean ± SD.

** CC, correlation coefficient. Bold values are significant *p* value.

A significant correlation was found between the Barratt score (impulsivity) and Young score (internet addiction) (*r* = 0.602 and *p* = 0.001). Furthermore, the inhibitory score indicated a weak correlation with internet addiction only in female subjects (*r* = 0.187 and *p* = 0.033) (Table 3).

**Table 3:** Correlation between age, Barratt score, Young score, and inhibition power status.

**Characteristics**		**Impulsivity**			**Inhibitory score**		
		**Total**	**Male**	**Female**	**Total**	**Male**	**Female**
Internet addiction	CC^*^	0.602	0.672	0.459	0.119	0.044	0.187
	*P*	**0.001**	**0.001**	**0.001**	0.050	0.620	**0.033**

* CC, correlation coefficient. Bold values are significant *p* value.

**Figure 1:**
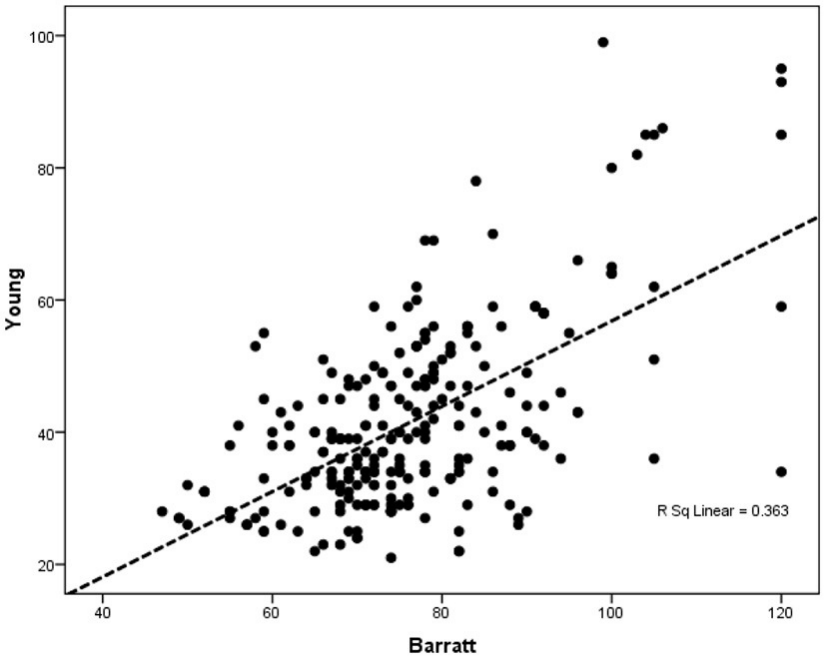
Multivariate regression analysis in determining the relationship between impulsivity and internet addiction adjusted for gender, age, and marital status. All samples included in the model and gender, age, and marital status was considered as covariates.

The findings based on multivariate regression analysis showed a significant correlation between the Barratt score (impulsivity) and Young score (internet addiction) in the presence of contextual indicators, including sex, age, and marital status (*p* < 0.0001) (Table 4; Figure 1).

**Table 4:** Multivariate regression analysis in determining the relationship between impulsivity and Internet addiction with the presence of gender, age, and marital status.

**Variables**	**Beta coefficient**	**Standard deviation**	***T*-score**	***P*-value**
Impulsivity	0.629	0.054	11.727	< 0.001
Sex	−2.692	1.396	−1.931	0.055
Age	−0.125	0.406	−0.309	0.758
Marital status	−1.339	2.170	−0.617	0.538

Independent: Young score, *R* squared = 0.363.

**Figure 2:**
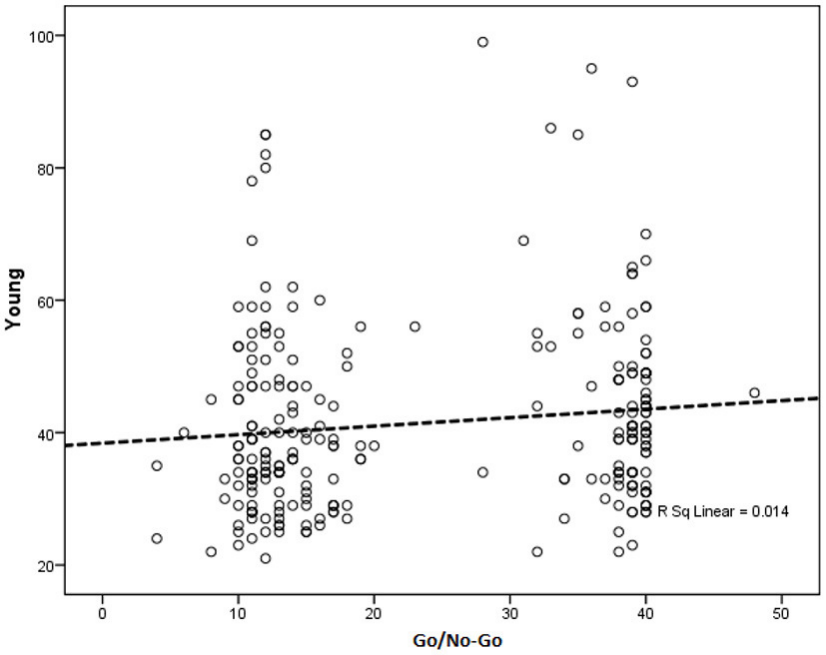
Multivariate regression analysis in determining the relationship between inhibition and Internet addiction adjusted for gender, age, and marital status. All samples included in the model and gender, age, and marital status was considered as covariates.

Considering inhibition power indicated no significant correlation between Young score Go/No-Go in the presence of contextual indicators, including sex, age, and marital status (*p* = 0.144) (Table 5; Figure 2).

**Table 5:** Multivariate regression analysis in determining the relationship between inhibition power and Internet addiction with the presence of gender, age, and marital status.

**Variables**	**Beta coefficient**	**Standard deviation**	***T*-score**	***P*-value**
Inhibition power	0.102	0.089	1.467	0.144
Sex	−4.889	1.717	−2.848	**0.005**
Age	−0.108	0.510	−0.211	0.833
Marital status	−0.831	2.720	−0.305	0.760

Independent: Internet addiction, *R* squared = 0.014. Bold values are significant *p* value.

## 5. Discussion

The present study investigated the relationship between internet addiction and impulsivity in a sample of Iranian medical students. The results revealed a significant direct correlation between impulsivity and internet addiction. Moreover, impulsivity was a predictor of internet addiction risk in the presence of pre-existing factors. These findings are in line with previous studies reporting that a higher impulsivity trait is more likely to engage in impulsive internet use. Therefore, Internet addiction might be considered an impulse control disorder.

According to our findings, the prevalence of internet addiction was 22.8% among medical students; 3.4% indicated high levels. Similarly, problematic internet use was reported by 21% of Spanish university students (28). Moreover, it was 19.9% of Indian college students (29). A recent meta-analysis study has reported the overall pooled prevalence of internet addiction by 34.53% among African high school and university students (30). In addition to the type of device and access level to the Internet in different countries, instruments assessing internet addiction and different cut-offs may illustrate a wide variety in the reported prevalence.

Based on our findings, impulsivity was associated with internet addiction. Furthermore, impulsivity was positively correlated with internet addiction, and the scores for impulsivity increased as the severity of internet addiction increased. A recent study has similarly indicated a positive relationship between these factors among university students (31). Another case-control study in Chinese adolescents indicated that subjects with Internet addiction exhibit more impulsive behaviors than controls. Moreover, they presented comorbid psychiatric disorders, probably associated with the psychopathology of Internet addiction (1). Consistent with these studies and others, these findings support the assumption that impulsivity is considered a risk marker of internet addiction, as individuals with internet addiction display an elevated tendency toward most impulsivity traits compared to non-Internet addiction.

Another finding of the present study indicated that addictive and impulsive traits were influenced by gender, as male subjects had higher mean in the Young and Barratt scores than females. Although we found that the correlation between internet addiction and impulsivity was slightly higher in male students than female subjects (*r* = 672 in males vs. *r* = 0.459 in females), it was not different between genders. On the other hand, the inhibition index was non-significantly higher in males than in females. However, internet addiction was significantly correlated with the restraint index in females, not males. In line with our results, a recent meta-analysis study has revealed a modulatory effect of gender on the association between restraint index and Internet addiction. This study has indicated that restraint indicator and Internet addiction was stronger among males than females. Similarly, the positive correlation between impulsivity and Internet addiction did not influence by gender (32). However, further studies should consider the mechanisms involved in gender regulatory function on inhibitory vs. impulsivity behaviors.

This study had some limitations that should be acknowledged. The sample size was small, and only one Medical university was included; thus, the generalization of the results may be limited. Moreover, the study did not include some confounding factors, including medications, drug dependency, and other psychiatric diseases. These factors can affect addictive and impulsive behaviors. As a cross-sectional design, it was also impossible to make conclusive statements about the temporal order between impulsivity traits and internet addiction. It is suggested that future longitudinal studies are designed with larger and more homogenous samples to overcome these limitations. Furthermore, examining the effect of the internet addiction withdrawal period on cognitive impulsivity may be another objective.

## 6. Conclusion

The present study found that the impulsivity trait is associated with Internet addiction. Furthermore, a significant positive correlation was observed between the severity of Internet addiction and the level of impulsivity. These findings may introduce Internet addiction as an impulse control disorder and impulsivity as a risk marker of Internet addiction.

## Data availability statement

The raw data supporting the conclusions of this article will be made available by the authors, without undue reservation.

## Ethics statement

The study was conducted by the Declaration of Helsinki and approved by the Ethics Committee at Golestan University of Medical Sciences IR.GOUMS.REC.1400.103. All participants were informed that participation is voluntary and reassured that responses would remain confidential. Informed written consent was also obtained from all participants filling in the questionnaires. Participants may withdraw from the trial at any point without any penalty and will not receive compensation for taking part. In the study, personal information about participants collected during the consent/data collection processes are stored securely. The patients/participants provided their written informed consent to participate in this study.

## Author contributions

MS: study concept and design, data curation, investigation, writing original draft, and writing—review and editing. ZA: data collection, methodology, project administration, writing original draft, and writing—review and editing. NS: writing the article and final approval of the article. AM: conceptualization, investigation, methodology, project administration, resources, and writing—review and editing. All authors contributed to the article and approved the submitted version.
